# Atopy Increases Risk of Psychotic Experiences: A Large Population-Based Study

**DOI:** 10.3389/fpsyt.2019.00453

**Published:** 2019-07-09

**Authors:** Marieke J.H. Begemann, Mascha M.J. Linszen, Janna N. de Boer, Wytske D. Hovenga, Shiral S. Gangadin, Maya J.L. Schutte, Iris E.C. Sommer

**Affiliations:** ^1^Cognitive Neurosciences, Department of Biomedical Sciences of Cells & Systems, University Medical Center Groningen, University of Groningen, Groningen, Netherlands; ^2^Department of Psychiatry, University Medical Center Utrecht, Utrecht, Netherlands

**Keywords:** psychotic experiences, hallucinations, delusions, atopic disorders, asthma, eczema, allergic rhinitis, immune

## Abstract

**Introduction:** Building upon the comorbidity between atopy and schizophrenia, we conducted a large cross-sectional, observational population-based study to examine if such associations also exist between atopic disorders (eczema, allergic rhinitis, and asthma) and nonclinical psychotic experiences.

**Methods:** We examined psychotic experiences in a Dutch population sample through an online survey (≥14 years of age). Participants filled out the Questionnaire for Psychotic Experiences, together with questions screening for atopic disorders (eczema, allergic rhinitis, and asthma). Prevalence rates were calculated; binary logistic regression was used to determine odds ratios (ORs) (age, gender, and years of education as covariates).

**Results:** We included 6,479 participants. Individuals diagnosed with one or more atopic disorders had an increased risk of psychotic experiences as compared with controls (OR = 1.26). Analysis of individual symptoms revealed an OR of 1.27 for hallucinations, whereas delusions only showed a trend. With each additional atopic disorder, the risk of psychotic experiences increased. This was also observed for hallucinations alone but not for delusions alone. Atopy was associated with hallucinations across all modalities (OR ranging from 1.19 to 1.40). These results did not appear to be driven specifically by any one of the atopic disorders.

**Conclusion:** In the largest population sample of adolescents and adults to date, we found that atopic disorders (asthma, eczema, and allergic rhinitis) increase the risk of psychotic experiences, in a dose–response fashion. These results provide further support for the role of immunological components in the predisposition for psychosis and can serve as a base for further research.

## Introduction

During the past years, the role of immunological factors [e.g., microglia, major histocompatibility complex (MHC), and complement molecules] in the pathogenesis of psychosis is supported by an increasing amount of data (1, 2). Speculation as to how immunological processes may be linked to the development of psychotic experiences has been fueled by epidemiological studies on schizophrenia patients and their relatives, finding altered prevalence rates of various autoimmune diseases such as celiac disease, autoimmune thyrotoxicosis, psoriasis, and pernicious anemia (3). For instance, a large Danish register-based study including 7,704 schizophrenia patients (and 192,590 controls) found that the incidence of autoimmune disorders was higher both in patients and their parents (4). Notably, a history of autoimmune disorder was related to a 45% increase in risk of schizophrenia (4).

A similar association has been shown for atopic disorders (i.e. a familial group of allergic disorders including asthma, allergic rhinitis, urticaria, and atopic dermatitis). A cross-sectional study in Taiwan by Chen and colleagues (44,187 schizophrenia patients; 132,748 controls) (5) observed a 1.3-fold increased risk of concurrent asthma in patients as compared with individuals without any psychiatric disease. Similarly, Weber et al. reported a relative high prevalence of asthma and eczema in schizophrenia patients (6). A longitudinal Danish register-based study (*n* = 808,559) found that individuals diagnosed with an atopic disorder earlier in life had an increased relative risk of 1.45 of developing schizophrenia in adulthood (7); their results were mainly driven by asthma (relative risk = 1.59). Interestingly, this association has even been observed across the psychosis continuum, as a population-based longitudinal study (*n* = 6,784) showed that children diagnosed with eczema and/or asthma had an increased risk of developing psychotic experiences in their adolescence (odds ratio [OR] = 1.44) (8).

To extend these findings, we conducted the first large-scale population-based study of atopic disorders and psychotic experiences in both adolescents and adults. We investigated whether atopy (defined here as diagnosed asthma, eczema, and allergic rhinitis) increased the risk of psychotic experiences (defined as hallucinations in four sensory modalities and nine delusion subtypes) in a sample of individuals aged 14+ years (*n* = 6,479).

## Materials and Methods

### Participants

The current study is part of a larger cross-sectional, observational project conducted in the Netherlands, entitled “Zie ik spoken” [“Do I see ghosts?”; for methodology, see Linszen et al. (9)]. Inclusion took place through the project’s website (www.zieikspoken.nl). The project was promoted between September 2016 and May 2017 through national media channels and several events. The current study included participants aged 14 years and over.

### Measures

#### Questionnaire for Psychotic Experiences

The Questionnaire for Psychotic Experiences (QPE) evaluates the presence and phenomenology of psychotic experiences, covering the full spectrum of hallucinations and delusions of any origin, severity, and duration (9, 10). As described by Linszen et al. (9), a self-survey version was used, addressing hallucinations in four sensory modalities and nine delusion subtypes. Screening questions evaluated the lifetime experience of a psychotic phenomenon. When answered affirmatively, follow-up questions regarding phenomenology were asked when this experience also occurred during the past week. Hallucinations were defined as a perception without a clear source from the environment. Delusions were considered as such if the participant reported being near to fully convinced of their truth. Prevalence rates were calculated by merging subtypes of hallucinations into one binary variable; a similar approach was used for the different delusion subtypes. The variable psychotic experiences were calculated by merging hallucinations and delusions.

#### Atopic Disorders

Screening questions addressed whether the participant ever suffered from asthma, eczema, or allergic rhinitis (described as hay fever) and, if so, whether the illness had been diagnosed by a doctor. Participants were divided into two different groups:

— *Atopy:* participants who reported one or more atopic disorder as diagnosed by a doctor [in line with previous register-based studies (5, 7)]. To evaluate a possible additive effect, the following subgroups were evaluated: 1) one atopic disorder; 2) two atopic disorders; 3) three atopic disorders. We also examined effects of the three atopic disorders individually.
— *Controls:* participants without any atopic disorder, including those individuals who reported having one or more atopic disorders without being diagnosed by a doctor.

### Procedure

**Figure 1:**
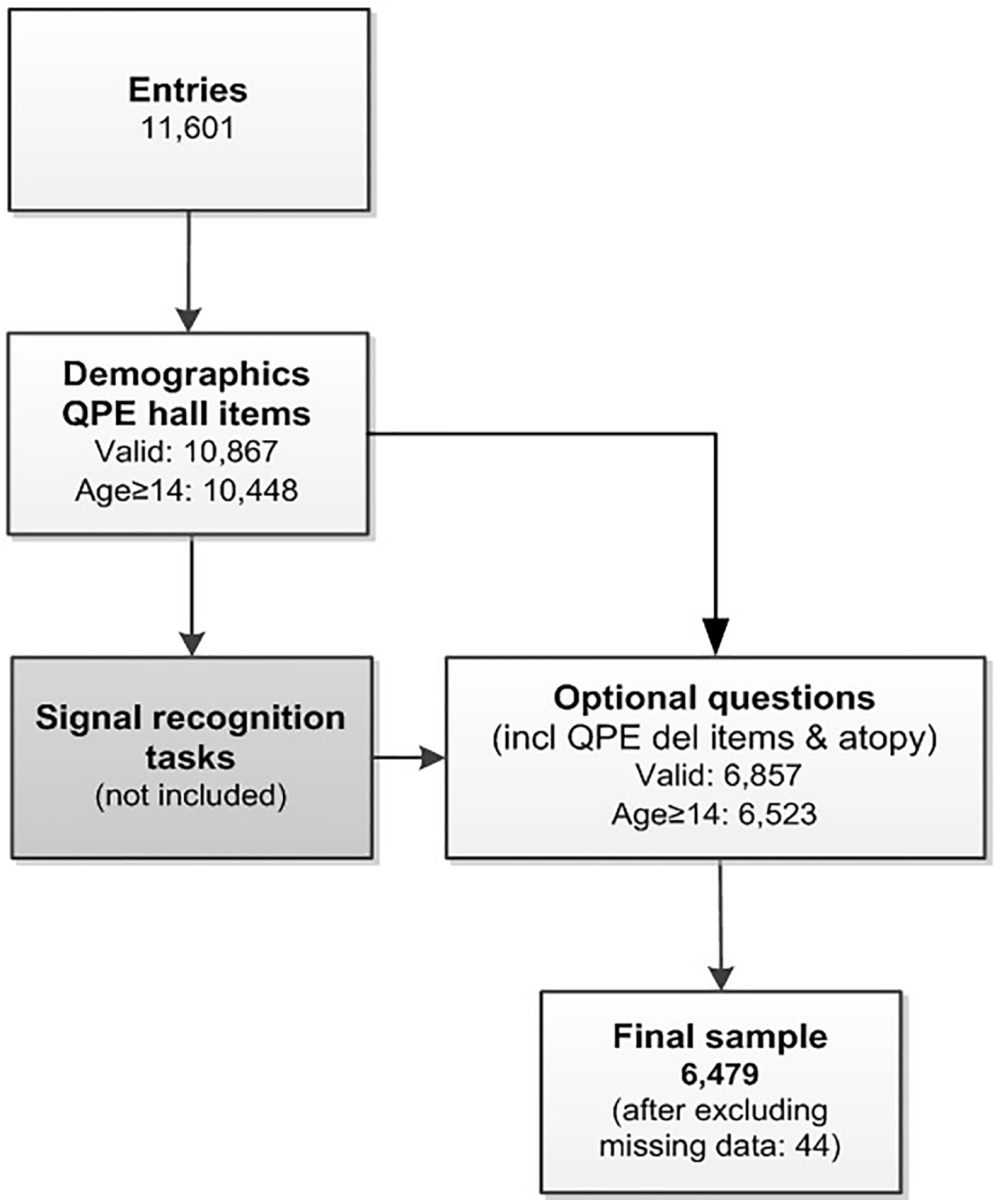
Flow chart of the study.

Participants submitted their data *via* the website https://zieikspoken.nl [11,601 entries, see flowchart in **Figure 1**; a detailed overview is provided by Linszen et al. (9)]. The Medical Research Ethics Committee of the University Medical Center Utrecht exempted this study from full review (local protocol number 16-408/C). All participants (including underaged subjects) confirmed their participation *via* the study website. Participants filled out demographic data (e.g., age, sex, handedness, highest level of education), followed by the QPE hallucination items. After two recognition tasks [results described by De Boer (11)], participants could progress to the QPE items on delusions and the questions evaluating atopic disorders (6,857 valid entries). The current sample was restricted by age (≥14 years), resulting in 6,479 participants with complete data.

### Statistics

Data were analyzed using IBM SPSS statistics (25.0). Demographics and prevalence rates of psychotic experiences were calculated for each group. Binary logistic regression was used to calculate ORs for psychotic experiences for the atopy (sub)group(s) as compared with the control group (age, gender, and years of education as covariates). We also evaluated the different subtypes of hallucinations and delusions as well as the individual atopic disorders (asthma, eczema, and allergic rhinitis).

## Results

### Baseline Group Comparisons

About one third (37.8%) of the sample (*n* = 6,479) reported having atopic disorders as diagnosed by a doctor (see **Figure 1** and **Table 1**). Compared with the control group, the atopy group included fewer males (*p* ≤ .001). Percentage of individuals with psychotic experiences within the different groups are presented in **Table 1**.

**Table 1:** Demographic characteristics of the study sample.

	No atopy (*n* = 4,040)	Atopy (*n* = 2,439)	*Asthma* (*n* = 954)	*Eczema* (*n* = 1,181)	*Allergic rhinitis* (*n* = 1,252)	*p* value (atopy vs. controls)^a^
**Male (%)**	34.0%	27.3%	26.7%	22.9%	29.2%	*p* < .001
**Age,** **mean (SD)**	36.8 (15.7)	36.5 (14.5)	35.1 (14.5)	37.0 (14.3)	36.5 (13.9)	*p* = .896
**Years of education, mean (SD)**	14.1 (2.1)	14.1 (2.1)	13.9 (2.1)	14.2 (2.0)	14.1 (2.0)	*p* = .593
**Psychotic experiences**	33.6%	39.6%	41.6%	38.4%	42.5%	
**Hallucinations**	31.2%	37.1%	39.2%	36.6%	39.9%	
*Auditory*	13.9%	18.2%	20.4%	17.5%	20.1%	
*Visual*	11.8%	14.0%	15.7%	13.2%	15.1%	
*Tactile*	10.1%	13.2%	13.4%	13.0%	14.1%	
*Olfactory*	9.0%	12.5%	13.4%	11.9%	13.3%	
**Delusions**	6.5%	7.7%	8.5%	6.8%	7.2%	
*Paranoia*	1.7%	2.3%	2.8%	2.1%	2.1%	
*Reference*	1.6%	1.8%	2.3%	1.5%	1.7%	
*Guilt*	1.0%	1.5%	1.9%	1.6%	1.3%	
*Control*	0.9%	0.7%	0.9%	0.8%	0.7%	
*Religious*	0.5%	0.7%	1.0%	0.5%	0.5%	
*Grandeur*	1.5%	2.1%	2.3%	1.7%	2.0%	
*Somatic*	0.8%	1.4%	1.8%	1.4%	1.1%	
*Capgras*	0.4%	0.5%	0.3%	0.4%	0.6%	
*Cotard*	0.1%	0.5%	0.2%	0.2%	0.2%	

a Gender: *χ*^2^; age and years of education: Mann–Whitney U test. n, sample size; SD, standard deviation.

### Atopy and Psychotic Experiences

Individuals with one or more atopic disorders had a significantly increased risk of psychotic experiences as compared with those without such a diagnosis, with an OR of 1.26 (95% CI: 1.14–1.41; *p* < .001). A similar OR was observed for hallucinations (OR 1.27; 95% CI: 1.14–1.42; *p* < .001), while the association with delusions bordered on significance (OR 1.21; 95% CI: 0.99–1.48; *p* = .054).

**Figure 2:**
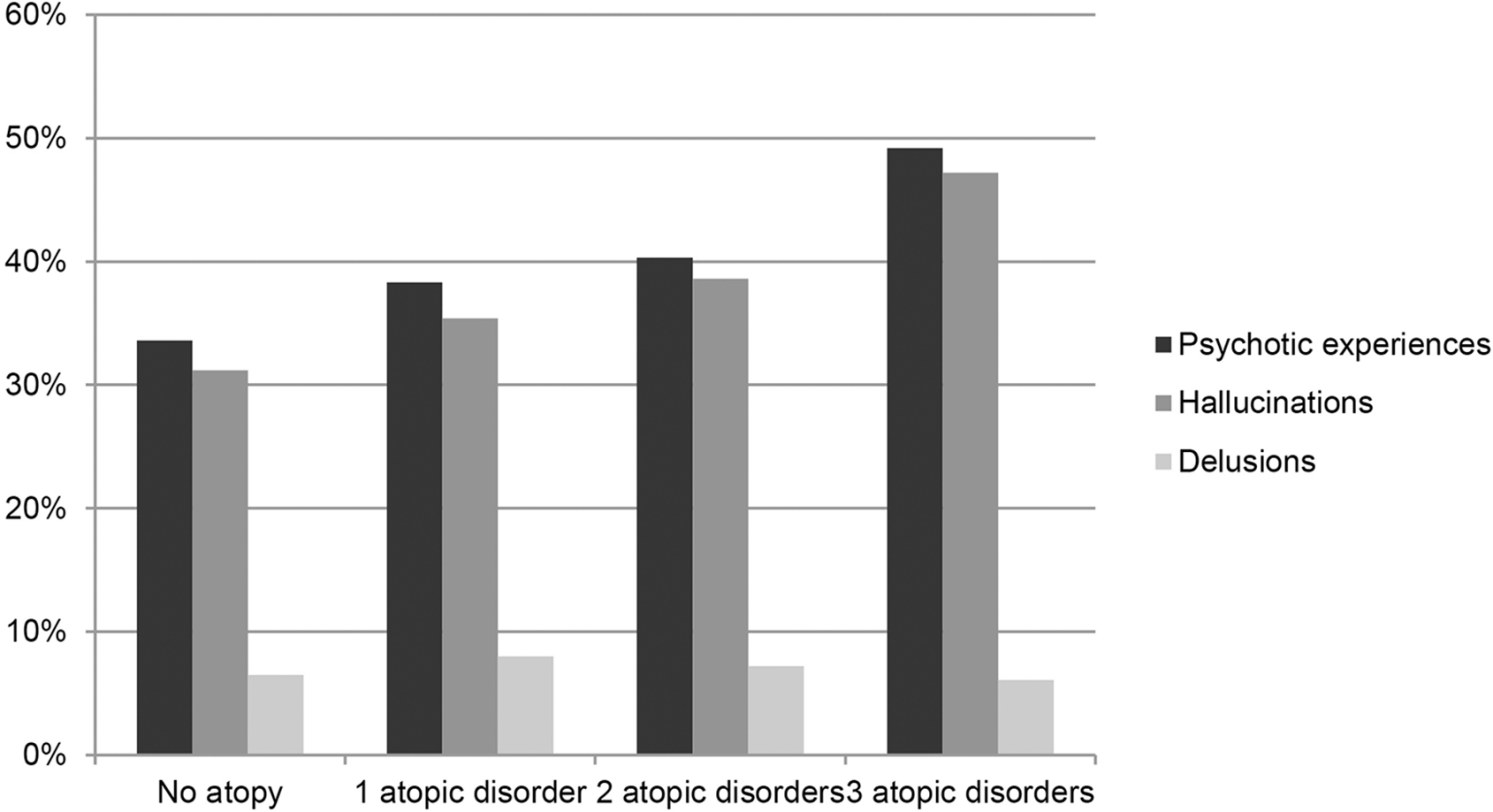
Percentage of individuals reporting psychotic experiences, grouped by the number of diagnosed atopic disorders.

We also evaluated the possible additive effect of multiple atopic disorders (**Figure 2**). With each additional diagnosis of atopic disorder, the odds for psychotic experiences increased (**Table 2**). This pattern was also observed for hallucinations but not for delusions.

**Table 2:** Odds ratios (ORs) for psychotic experiences per number of diagnosed atopic disorders.

	1 atopic disorder (*n* = 1,688)	2 atopic disorders (*n* = 554)	3 atopic disorders (*n* = 197)
Psychotic experiences	**1.20** ***p* = .004** (1.06–1.35)	**1.33** ***p* = .003** (1.10–1.60)	**1.80** ***p* < .001** (1.34–1.42)
Hallucinations	**1.18** ***p* = .008** (1.05–1.34)	**1.38** ***p* = .001** (1.15–1.67)	**1.85** ***p* < .001** (1.38–2.48)
Delusions	**1.26** ***p* = .037** (1.02–1.57)	1.17 *p* = .376 (0.83–1.66)	0.93 *p* = .810 (0.51–1.70)

The group without (diagnosed) atopic disorders was used as reference (OR = 1.00). ORs adjusted for age, gender, and years of education (OR, 95% confidence interval). Raw percentages (noted for the 1 atopic disorder, 2 atopic disorders, and 3 atopic disorders groups): psychotic experiences 38.3, 40.3, and 49.2%; hallucinations 35.4, 38.6, and 47.2%; delusions 8.0, 7.2, and 6.1% (also see **Figure 2**).

### Hallucination and Delusion Subtypes

Compared to controls, atopy [≥ 1 diagnosed atopic disorder(s)] was associated with increased odds across all hallucination modalities with ORs ranging between 1.19 and 1.40 (OR for visual hallucinations was trend-level significant after Bonferroni correction), see **Table 3**. Atopy was not associated with any of the delusion subtypes.

**Table 3:** Associations between specific psychotic experiences and atopy, including the individual atopic disorders.

	Atopy (*n* = 2,439)	*Asthma* (*n* = 954)	*Eczema* (*n* = 1,181)	*Allergic rhinitis* (*n* = 1,252)
Psychotic experiences	**1.26** ***p* < .001** (1.14–1.41)	**1.32** ***p* < .001** (1.14–1.53)	**1.20** ***p* = .009** (1.05–1.38)	**1.46** ***p* < .001** (1.28–1.67)
Hallucinations	**1.27** ***p* < .001** (1.14–1.42)	**1.34** ***p* < .001** (1.15–1.55)	**1.24** ***p* = .002** (1.08–1.43)	**1.47** ***p* < .001** (1.28–1.68)
*Auditory*^a^	**1.36** ***p* < .001** (1.19–1.57)	**1.51** ***p* < .001** (1.26–1.82)	**1.31** ***p* = .003** (1.10–1.57)	**1.58** ***p* < .001** (1.34–1.87)
*Visual*^a^	1.19 *p* = .026 (1.02–1.38)	**1.32** ***p* = .006** (1.08–1.62)	1.10 *p* = .339 (0.91–1.34)	**1.31** ***p* = .003** (1.10–1.58)
*Tactile*^a^	**1.33** ***p* < .001** (1.14–1.56)	1.29 *p* = .022 (1.04–1.60)	**1.31** ***p* = .010** (1.07–1.60)	**1.47** ***p* < .001** (1.21–1.78)
*Olfactory*^a^	**1.40** ***p* < .001** (1.19–1.65)	**1.51** ***p* < .001** (1.21–1.87)	**1.32** ***p* = .009** (1.08–1.63)	**1.53** ***p* < .001** (1.26–1.87)
Delusions	1.21 *p* = .054 (0.99–1.48)	1.30 *p* = .051 (0.99–1.69)	1.07 *p* = .594 (0.83–1.40)	1.14 *p* = .304 (0.89–1.47)
*Paranoia*^b^	1.31 *p* = .142 (0.91–1.87)	1.55 *p* = .059 (0.98–2.43)	1.22 *p* = .402 (0.77–1.94)	1.22 *p* = .395 (0.77–1.93)
*Reference*^b^	1.17 *p* = .422 (0.80–1.73)	1.45 *p* = .137 (0.89–2.38)	0.96 *p* = .876 (0.56–1.63)	1.07 *p* = .794 (0.65–1.76)
*Guilt*^b^	1.60 *p* = .041 (1.09–2.53)	1.87 *p* = .031 (1.06–3.29)	1.71 *p* = .059 (0.98–2.99)	1.40 *p* = .264 (0.78–2.52)
*Control*^b^	0.87 *p* = .644 (0.49–1.55)	1.09 *p* = .828 (0.52–2.27)	0.93 *p* = .852 (0.44–1.95)	0.84 *p* = .646 (0.40–1.76)
*Religious*^b^	1.43 *p* = .262 (0.76–2.68)	2.02 *p* = .069 (0.95–4.29)	1.05 *p* = .913 (0.42–2.62)	0.91 *p* = .843 (0.37–2.26)
*Grandeur*^b^	1.44 *p* = .060 (0.99–2.11)	1.60 *p* = .062 (0.98–2.63)	1.23 *p* = .437 (0.73–2.05)	1.39 *p* = .168 (0.87–2.24)
*Somatic*^b^	1.67 *p* = .039 (1.03–2.70)	2.11 *p* = .013 (1.17–3.80)	1.74 *p* = .072 (0.95–3.19)	1.40 *p* = .289 (0.75–2.64)
*Capgras*^b^	1.24 *p* = .573 (0.59–2.61)	0.70 *p* = .567 (0.20–2.40)	1.07 *p* = .896 (0.39–2.93)	1.64 *p* = .252 (0.70–3.84)
*Cotard*^b^	1.72 *p* = .395 (0.49–5.96)	1.72 *p* = .521 (0.33–8.92)	1.50 *p* = .634 (0.39–7.83)	2.05 *p* = .327 (0.49–8.64)

The group without (diagnosed) atopic disorders was used as reference (OR = 1.00). ORs adjusted for age, gender, and years of education (OR, 95% confidence interval).

a Alpha level was Bonferroni-corrected for hallucination subtypes (.05/4 = .0125)

b Alpha level was Bonferroni-corrected for delusion subtypes (.05/9 = .0056).

### Asthma, Eczema, and Allergic Rhinitis

Significant odd ratios for psychotic experiences were found for allergic rhinitis, asthma, and eczema as compared with those without atopic disorders (**Table 3**). The ORs were highest for allergic rhinitis (1.46), followed by asthma (1.32), and eczema (1.20). Results were similar for hallucinations, while the associations with delusions were not significant for any of the individual atopic disorders (asthma bordered on significance, 1.30). Regarding hallucination subtypes, all associations were significant except between asthma and tactile hallucinations and between eczema and visual hallucinations, which did not survive Bonferroni correction (**Table 3**). None of the delusion subtypes were significantly associated with asthma, eczema, or allergic rhinitis.

## Discussion

To our knowledge, this is the largest population-based study investigating the association between atopic disorders and psychotic experiences in an adolescent and adult sample. We observed an increased risk of psychotic experiences in individuals with atopic disorders as compared with those without, specifically for hallucinations. We found a dose–response relationship, with each additional atopic disorder diagnosis increasing the odds for psychotic experiences. This was also observed for hallucinations alone but not for delusions alone. Atopy was associated with hallucinations across all modalities, while the odds for delusions were not significant. These results did not appear to be driven specifically by allergic rhinitis, asthma, or eczema.

In comparison with findings of Khandaker and colleagues on childhood atopy and psychotic experiences in adolescence (8), we also found an increased risk of psychotic experiences in individuals with atopic disorders, both dichotomous and in a dose–response relationship. We replicated their finding that asthma and eczema are specifically associated with psychotic experiences, and extended these findings by also reporting higher odds for allergic rhinitis. In contrast to Khandaker (8), we found that atopy was associated with hallucinations across four sensory modalities, instead of auditory hallucinations alone (although the result for visual hallucinations was trend-level significant after correcting for multiple comparisons). While Khandaker found associations between atopy and two delusion subtypes (8), our results did not reach significance, which could be due to the limited number of reported delusion subtypes (percentages ranging between 0.1 and 2.8%).

Our findings are in accordance with previous studies reporting the high incidence of atopic disorders in schizophrenia patients by Chen and colleagues (5) and the increased risk of schizophrenia in individuals with atopic disorders by Pedersen et al. (7). Notably, both studies evaluated four different atopic disorders (asthma, allergic rhinitis, urticaria, and eczema) and found that their results were mainly driven by asthma. The high comorbidity between asthma and schizophrenia has also been reported by Weber et al. (6). This disorder-specific association was not evident in our sample, as the increased risk of psychotic experiences was observed for asthma, eczema, and allergic rhinitis. Moreover, our finding for allergic rhinitis does not corroborate the observation by Chen and colleagues (5), who observed that the risk for diagnosed allergic rhinitis was decreased in schizophrenia patients. However, Pederson et al. did report increased odds for allergic rhinitis but only when combined with urticaria and eczema (7).

In line with the continuum hypothesis of psychosis (12) describing psychotic symptoms along a continuous range (from isolated, nonclinical symptoms to those occurring in the context of psychotic disorders), our results contribute to the previously found associations between autoimmune disorders, atopy and schizophrenia (3–8). Current findings also corroborate previous genetic and biomedical studies that have indicated the involvement of immune-mediated pathways in the development of psychosis (1, 2). Genome-wide association studies have identified robust schizophrenia-associated risk loci involved in adaptive immunity (CD19 and CD20 B-lymphocytes) and the MHC region on chromosome six (1, 2, 13). The MHC region is best known for its involvement in antigen presentation and inflammatory mediators. It has also been suggested that asthma and schizophrenia may share genetic susceptibility (8, 14). Diverse immune alterations have been found in patients with a psychotic disorder, indicating inflammation of the central nervous system (1, 3). Cross-sectional studies have linked inflammatory parameters in the blood, including C-reactive protein as well as interleukin-6 and interleukin-8, to severity of negative symptoms and cognitive performance in schizophrenia patients (15). Furthermore, as visualized with positron electron tomography, an increased number of activated microglia cells has been found in the brains of patients with recent-onset psychosis and those at ultra-high risk of psychosis (16, 17). Excessive microglial activity potentially provides a route by which an increased pro-inflammatory state in the brain may contribute to the gray matter thinning and synapse loss observed in schizophrenia patients (18, 19). Future research should further evaluate the complex interplay between mediating factors that are relevant for both psychosis and atopy, including genetics and environmental factors such as urbanicity and childhood trauma (20–23). Importantly, more insight in the immunological components of psychosis provides new leads to improve treatment options, with anti-inflammatory drugs being viewed as potential candidates for new augmentation therapies for at least a subset of patients (24).

### Strengths and Limitations

A major strength of this study is the large number of participants. Online questionnaires are suitable to evaluate common phenomena and their risk factors in large samples, which can reveal subtle associations. Given the anonymity of participation, they are also suitable for stigmatized topics such as psychotic experiences.

One important limitation is that online surveys are more prone to participation bias and skewed population samples, which prompted us to include demographic variables as covariates in our analyses (25, 26). In addition, the choice to participate is influenced by individual interests and preferences that could have contributed to the relatively high percentages of hallucinations we observed. These percentages should hence be interpreted with caution [for an elaboration on this point, see Refs. (9, 11)]. Furthermore, similar to the Khandaker et al.’s (8) study, we based the presence of atopic disorders on these self-reported data. Although we specifically asked whether these atopic disorders had been diagnosed by a doctor, these data could not be verified by making use of health records. A large systematic review by Pols et al. (27) compared self-reported prevalences from population-based studies with clinician-diagnosed prevalences in general practice (children aged 0–18 years, in the UK and the Netherlands) and concluded that the lifetime prevalence of asthma, eczema, and allergic rhinitis was lower in general practice. They also noted that individual estimates varied widely in both population-based and general practice studies. Our population-based lifetime prevalence rates of 14.7% for asthma, 18.2% for eczema, and 19.3% for allergic rhinitis were relatively low compared to those found by Pols et al. (asthma: population 19.1–35.6% vs. general practice 4.2–22.9%; eczema: population 16.5–27.1% vs. general practice 7.2–36.5%; allergic rhinitis: population 18.3–47.7% vs. general practice 1.0–11.4%). Importantly, the ORs we found for atopic disorders increasing the risk for psychotic experiences were comparable with those reported by previous studies using national-register data (5, 7). We also performed additional sensitivity analyses, as 16.8% of our control sample reported an atopic disorder that was not diagnosed by a doctor, which may have introduced some false negatives. Excluding these individuals altogether (resulting in a sample of *n* = 5,800) gave similar findings with overall higher ORs (atopy versus controls: psychotic experiences OR 1.34; hallucinations OR 1.34; delusions OR 1.24). Our trend association between atopy and delusions now reached significance (*p* = .043).

As mentioned above, various factors could mediate the found associations between atopy and risk for psychotic experiences. For example, our dataset did not include measurements on urbanicity. However, two previous studies that further adjusted their estimates for degree of urbanization did not find a significant change in their results without this correction (5, 7). Furthermore, it is important to note that, although our findings were statistically significant, the percentage of reported psychotic experiences was only a few percent higher in participants with atopic disorders as compared with that in the control group. However, the effects were evident in a sample taken from the general population. Our findings indicate that atopic disorders are a risk factor even for the development of nonclinical psychotic experiences. This supports the proposition that the immunological pathway may constitute an important common underlying pathway in the development of psychotic experiences across the psychosis continuum, both in healthy individuals and in patients with a psychotic disorders (8, 12).

### Conclusion

In the largest population-based study in adolescents and adults to date, we found that atopic disorders (asthma, eczema, and allergic rhinitis) increased the risk of psychotic experiences in a dose–response fashion. These results provide further support for the involvement of immunological processes in the pathophysiology of psychosis.

## Ethics Statement

Given the observational nature of this study, the medical ethics committee of the University Medical Center Utrecht exempted the project from further review.

## Author Contributions

Writing manuscript: MB. Preparation of the database: ML, JB, WH, and MS. Revising manuscript content: ML, JB, SG, IS. Approving final version of the manuscript: all authors.

## Conflict of Interest Statement

The authors declare that the research was conducted in the absence of any commercial or financial relationships that could be construed as a potential conflict of interest.
